# The Role of Statins in Disease Modification and Cardiovascular Risk in Rheumatoid Arthritis

**DOI:** 10.3389/fmed.2018.00024

**Published:** 2018-02-08

**Authors:** Stergios Soulaidopoulos, Elena Nikiphorou, Theodoros Dimitroulas, George D. Kitas

**Affiliations:** ^1^4th Department of Internal Medicine, Hippokration University Hospital, Aristotle University of Thessaloniki, Thessaloniki, Greece; ^2^Department of Academic Rheumatology, King’s College London, London, United Kingdom; ^3^Department of Rheumatology, Whittington NHS Health, London, United Kingdom; ^4^Arthritis Research UK Centre for Epidemiology, University of Manchester, Manchester, United Kingdom; ^5^Department of Rheumatology, Dudley Group NHS Fountation Trust, Dudley, United Kingdom

**Keywords:** statins, rheumatoid arthritis, cardiovascular risk, lipid lowering action, anti-inflammatory effect

## Abstract

Rheumatoid arthritis (RA) is an autoimmune, inflammatory disorder associated with excess cardiovascular morbidity and mortality. A complex interplay between traditional risk factors (dyslipidemia, insulin resistance, arterial hypertension, obesity, smoking) and chronic inflammation is implicated in the development of premature atherosclerosis and consequently in the higher incidence of cardiovascular events observed in RA patients. Despite the acknowledgment of elevated cardiovascular risk among RA individuals, its management remains suboptimal. While statin administration has a crucial role in primary and secondary cardiovascular disease prevention strategies as lipid modulating factors, there are limited data concerning the precise benefit of such therapy in patients with RA. Systemic inflammation and anti-inflammatory treatments influence lipid metabolism, leading to variable states of dyslipidemia in RA. Hence, the indications for statin therapy for cardiovascular prevention may differ between RA patients and the general population and the precise role of lipid lowering treatment in RA is yet to be established. Furthermore, some evidence supports a potential beneficial impact of statins on RA disease activity, attributable to their anti-inflammatory and immunomodulatory properties. This review discusses existing data on the efficacy of statins in reducing RA-related cardiovascular risk as well as their potential beneficial effects on disease activity.

## Introduction

Rheumatoid arthritis (RA) imparts a significant risk for cardiovascular disease (CVD)-related morbidity and mortality (1, 2). It is well-established that accelerated atherosclerosis and vascular dysfunction in the setting of RA are the result of a complex interplay between traditional CVD risk factors, such as dyslipidemia (3), insulin resistance (4), hypertension (5), limited physical activity (6), and obesity (7), and RA-related characteristics including chronic high grade inflammation and autoimmune activation (8, 9). The recognition of the pivotal role of chronic inflammation in the pathogenesis of accelerated atherosclerosis in RA emphasizes the need for CVD risk management in these patients, to include both inflammation control and modification of traditional CVD risk factors. Therefore, assessment of CVD risk in patients with RA in routine clinical practice is highly recommended (10). However, the proposed CVD risk assessment scores designed for the general population seem to be insufficient in the case of RA patients, leading to an underestimation of CVD risk and, consequently, suboptimal CVD prevention (11).

Dyslipidemia constitutes the leading factor for cardiovascular events in the general population, making it the key target of current primary and secondary CVD prevention strategies (12). In this respect, several randomized clinical trials have firmly demonstrated the beneficial effect of statins, as lipid lowering agents on CVD risk management in many populations (13). However, the precise benefit of lipid lowering treatment in RA remains unclear. The variable prevalence of dyslipidemia in RA as well as the disproportionate association between lipid levels and disease activity, unrepresentative of the actual CVD risk, complicates the setting of low-density lipoprotein cholesterol (LDL-C) treatment targets and the choice of those patients eligible for statin therapy (14–17). Interestingly, experimental and clinical data support that statins may also provide anti-inflammatory and immunomodulatory benefits in RA, due to their pleiotropic therapeutic effects (18).

Cardiovascular events remain the major cause of death in patients with RA, urging a reassessment of the current approach to CVD risk management. Although many lines investigating this topic have been written, the exact benefit from statin treatment in RA needs further clarification. The scope of this manuscript is to undertake a narrative review of existing literature on the efficacy of statins in CVD prevention in RA, to discuss existing and future therapeutic targets arising from their pleiotropic pharmacological properties while addressing knowledge gaps and future research.

## Methods

A literature search was conducted using the online databases MEDLINE/PUBMED and EMBASE from January 2000 until July 2017 for original research articles and review articles investigating the effect of statin treatment in patients with RA. Data concerning both CVD management and the impact on several aspects of RA were collected. The combination of the following terms was used to identify relevant publications: statins OR atorvastatin OR simvastatin OR rosuvastatin OR HMG-CoA reductase inhibitor OR cardiovascular disease OR hypercholesterolemia OR dyslipidemia OR cholesterol AND rheumatoid arthritis. We also reviewed the literature for cited articles relevant to the subject in articles identified through the review, to ensure that we did not miss important research data. Full journal articles and published abstracts in English language were included. We used the above terms in ClinicalTrials.gov to search for any recently completed or ongoing relevant pharmaceutical research. Conference proceedings, not accessible abstracts, case reports, or articles not in English, were excluded (19).

## Statins and CVD Markers

The reduction of LDL-C by statins leads to a significant decrease in major cardiovascular events in patients at either high or low risk for CVD (20, 21). Such observations in the general population provide the rationale for treating RA patients with statins aiming to reduce the CVD burden (22). On top of the lipid lowering effect, other properties of statins, such as the stabilization and regression of atherosclerotic plaques and the limitation of LDL-C oxidation, could theoretically contribute to the prevention of primary atherosclerosis in RA (23, 24). Lipid profile alterations, displaying an inverse correlation between total cholesterol (TC) levels and disease activity, raise some questions about the exact value of lipid lowering treatment in RA (25). High level inflammation on the one hand and potent antiinflammatory disease-modifying antirheumatic drugs (DMARDs) on the other have significant contrasting effects on lipid levels and could modify the lipid lowering effectiveness and consequent CVD risk reduction impact of statins in RA (26, 27).

### Angioprotective Effect

Several clinical studies have attempted to investigate statin benefits in RA patients utilizing non-invasive assessments of vascular function and morphology. The need for early detection of pathological processes in vasculature has led to the development of non-invasive tests which now constitute valuable tools for cardiovascular risk monitoring. The flow-mediated dilatation (FMD) test provides an ultrasonic assessment of nitric oxide-dependent vascular endothelial function, which is disturbed in atheromatous vessels (28). Arterial stiffness, showing an increase in early atheromatosis, can accurately be assessed with aortic pulse wave velocity (PWV) and Augmentation Index (AIx) (29, 30), parameters that are considered to be independent predictors of major cardiovascular events (31). Vascular function and morphology assessed by these markers is impaired in RA relative to healthy controls even in early stages of RA, reflect in the elevated cardiovascular risk (32, 33).

In a trial of 20 RA patients, administration of simvastatin 40 mg/day significantly improved FMD compared with placebo (34). Notably, a higher FMD was observed in patients with higher inflammatory status. A lower dose of simvastatin also induced a parallel reduction in both FMD and aortic PWV (35). A 12-week atorvastatin treatment in the dose of 20 mg/day induced a 12% reduction in arterial stiffness, evaluated with AIx (36). The greatest reductions of AIx occurred in those patients with higher disease activity scores at baseline. Furthermore, it was demonstrated that, in RA patients, atorvastatin had a positive impact on novel serum markers of endocrine function of arterial wall, such as adiponectin, along with a collateral improvement of FMD (37).

The effect of intensive lipid lowering treatment was evaluated in the ROsuvastatin in Rheumatoid Arthritis and Ankylosing Spondylitis study (38, 39). A regression of carotid plaques, inversely associated with disease activity during the study period, was demonstrated after 18 months of increasing dosage of rosuvastatin, to the upper limit of 40 mg/day until LDL-C levels below 70 mg/dL were achieved, highlighting once again the angioprotective properties of statins. In contrast, no significant reduction in carotid intima–media thickness (IMT) and AIx was recorded in 50 RA patients with low-disease activity, randomized to take 10 mg of rosuvastatin or placebo for 12 months (40).

In the context of biomarkers of endothelial activation, the Trial for Atorvastatin in Rheumatoid Arthritis (TARA) included 116 RA patients, randomized to receive 40 mg atorvastatin or placebo additionally to the existing disease-modifying therapy. A significant improvement in intercellular adhesion molecule 1 and fibrinogen levels was observed in the atorvastatin group after six months of treatment (41).

Taking it all into account, statins seem to have a favorable effect on structural, morphological and biochemical parameters of vascular function in RA patients, particularly those with higher disease activity (Table 1). However, the confirmation of these findings in larger and better controlled studies, as well as hard cardiovascular end-point trials, is warranted before specific recommendations can be made for their use in the routine clinical setting.

**Table 1:** Summary of the studies assessing the impact of statins on vascular markers.

Reference	Population	Intervention	Parameter assessed	Findings
McCarey et al. (TARA) (41)	116 RA patients	40 mg atorvastatin or placebo for 6 months	– Endothelial activation – Disease activity Lipid levels	Decrease in: – Plasma viscosity, fibrinogen, soluble intercellular adhesion molecule 1, IL-6 – DAS28 score – CRP – TC, LDL, VLDL, TGL

Hermann et al. (34)	20 RA patients	40 mg simvastatin for 4 weeks followed by 4 weeks of placebo or vice versa	– Endothelial function – Lipid levels	– FMD improvement in statin group – Decrease in LDL, TC, apoB, oxLDL, oxLDL/LDL ratio

Maki-Petaja et al. (35)	20 RA patients	20 mg simvastatin or 10 mg ezetimibe for 6 weeks	– Disease activity – Endothelial function – Arterial stiffness – Blood pressure – Inflammatory markers – Lipid levels	– Decrease in DAS28 score – Increase of FMD – Decrease in APWV – NS change in AIx – NS change in arterial pressure – Decrease in CRP and ESR in both groups – Decrease in TC, LDL, oxLDL

Van Doornum et al. (36)	29 RA patients	20 mg atorvastatin for 12 weeks	– Arterial stiffness – Inflammatory markers – Disease activity – Lipid levels	– Decrease in AIx – NS change in CRP and ESR – NS change in DAS28 – Decrease in TC and LDL

El-Barbary et al. (37)	30 RA patients–10 controls	15 MTX + prednisone—15 MTX + prednisone + 40 mg atorvastatin for 4 months	– Disease activity – Lipid profile – Oxidative stress – Inflammatory mediators – Endothelial function	In the atorvastatin group: – Greater decrease in DAS28 – Decrease in TC, TG, LDL – Increase in HDL – Decrease in serum malondialdehyde – Greater decrease in TNF-α Improvement of resistin, adiponectin, and FMD

Rollefstad et al. (38) Ikdahl et al. (39)	55 RA–21 AS–10 psoriatic arthritis patients—all with carotid plaques	Rosuvastatin until LDL ≤ 70 mg/dl—18 months	– Atheromatic plaques – Lipid targets – Arterial stiffness – Blood pressure	– SS regression of carotid plaques – Decrease in PWV and AIx – Decrease in blood pressure – Reduction of LDL – NS linear correlation between plaque regression and LDL decrease

Tam et al. (40)	50 RA patients	10 mg rosuvastatin or placebo for 12 months	– Carotid atheromatosis – Arterial stiffness – Myocardial oxygen supply – Lipid levels – Disease activity – Inflammatory markers	– NS change in CIMT – NS change in AIx – Improvement of subendocardial viability ratio – Decrease in TC, LDL, apoB – Decrease in DAS28 – NS Change in CRP, ESR

*RA, rheumatoid arthritis; IL-6, interleukin-6; DAS28, disease activity score 28; CRP, C-reactive protein; TC, total cholesterol; LDL, low-density lipoprotein; VLDL, very low-density lipoprotein; FMD, flow-mediated dilatation; apoB, apolipoprotein B; oxLDL, oxidized LDL; aPWV, aortic pulse wave velocity; NS, non significant; AIx, Augmentation Index; ESR erythrocyte sendimentation rate; MTX, methoteraxate; HDL, high-density lipoprotein; TNF-α, tumor necrosis factor-α; AS, ankylosing spondylitis; SS, statistical significant; CIMT, carotid intima–media thickness*.

### Lipid Lowering and Antioxidative Effect

Several factors seem to influence the lipid profile of RA patients, including inflammatory disease activity, antirheumatic drugs, and reduced physical activity (16). Despite the demonstrated excessive premature atherosclerosis potentiated by systemic inflammation, some evidence supports a paradoxical decrease of lipid levels in untreated, active RA (42). Given that TC levels are inversely correlated with current risk for CVD, the exact value of an additional lipid lowering effect remains questionable. However, a decrease in TC cannot describe the total lipid alterations characterizing RA. The observed fall in TC levels does not seem to be as marked as that seen in high-density lipoprotein cholesterol (HDL-C) levels and this leads to an increased atherogenic index (TC:HDL ratio), exposing RA patients to higher risk of atherosclerosis (43). Moreover, other lipid components with high predictive value for CVD, such as lipoprotein(a) and apolipoprotein B, are elevated in RA, while the relation between LDL-C and high disease activity remains unclear (44).

An inverse correlation between lipid concentrations and inflammatory markers in RA patients treated with non-biological DMARDS has been observed. While methotrexate therapy combined with prednisolone has been associated with a significant increase in TC and HDL-C levels, a significant decrease in the atherogenic ratio of TC/HDL-C ratio along with a response to treatment has been recorded (45). Similarly, tumor necrosis factor-α (TNF-α) inhibitors have been found to increase TC and HDL-C without affecting LDL-C levels and the atherogenic ratio (46). On the other hand, a significant rise in LDL-C levels with conflicting results regarding the effect on the atherogenic ratio and the net impact on CVD risk has been documented in RA patients treated with the interleukin-6 (IL-6) receptor inhibitor tocilizumab (47, 48). A *post hoc* analysis of 4,655 patients included in tocilizumab trials found that postbaseline initiation of statins was related to a gradual decrease and stabilization of LDL-C after two years of treatment, in contrast to the patients not receiving statins, where a significant increase in lipid levels was observed (49). Despite the need for further evaluation, these findings may support the administration of statins for the management of unfavorable cholesterol changes attributed to treatment with specific classes of biologic DMARDs.

Existing data demonstrate that statins maintain their lipid lowering effect in patients with RA and active inflammation. The reduction of LDL-C and TC levels is combined with a concurrent decrease in C-reactive protein (CRP) levels (41). Lipid lowering properties of statins remain even in the presence of drugs with an opposite effect on lipid metabolism, such as corticosteroids (50). Conducting a sufficient evaluation of cardiovascular risk in patients with inflammatory joint disease using the systematic coronary risk evaluation (SCORE) (51), 165 RA patients with a SCORE of 5% or greater, received lipid lowering therapy as part of either primary or secondary CVD protection (52). Statin treatment was adjusted, until at least two lipid targets were reached. Overall, statin intervention proved 92.1% successful in achieving lipid goals. Low doses of atorvastatin, 5 and 10 mg/day, in combination with proper control of chronic inflammation and disease activity, were also effective in significantly decreasing lipid levels in 52 RA patients diagnosed with dyslipidemia (53). Besides a reduction of TC and LDL-C levels, 10 mg of rosuvastatin treatment for 12 months resulted in significantly lower apolipoprotein B levels compared to placebo (40).

The oxidized particles of LDL-C, known as oxidized LDL (oxLDL), are recognized as crucial factors in the pathogenesis of atherosclerosis, inducing endothelial dysfunction through stimulation of monocyte infiltration and smooth cell proliferation, impairment of endothelial signaling and generation of reactive oxygen species (54). Atherogenic oxLDL are elevated in RA and correlate with disease activity (55). Interestingly, simvastatin 40 mg/day for 4 weeks led to a reduction of oxLDL/LDL ratio, suggesting a decrease in the formation of reactive oxygen species (34).

Controversial data were presented from a retrospective analysis, addressing an adverse relation between markers of inflammation and the likelihood οf achieving LDL therapeutic targets in RA subjects (56). The precise impact of lipid lowering treatment in RA-related cardiovascular risk can only be evaluated in controlled, long-term, and hard end-point trials. Especially for those subjects with low cholesterol values due to active inflammation, it remains to be determined whether lower cholesterol levels correspond to significant cardiovascular benefit. Nevertheless, up to 25% of RA patients receive suboptimal treatment according to the estimated lipid-associated CVD risk and current guidelines for the general population (17).

### Primary and Secondary Prevention

While several reports highlight the improvement of various CVD markers and effectiveness in dyslipidemia management after statin administration in RA, comprehensive assessments on the statins’ potentials on CVD prevention can be obtained through controlled trials with “hard” cardiovascular end points. Aimed at assessing the hypothesis of atorvastatin’s superiority to placebo, the TRACE-RA trial (*n* = 2,986, median follow-up of 2.53 years) indicates a 34% reduction of major cardiovascular events in the statin group compared to placebo (57). The demonstrated benefit did not reach statistical significance [hazard ratio (HR): 0.66, 95% confidence interval (CI): 0.40–1.11, *p* = 0.119] likely to be secondary to the study’s premature termination due to the unexpectedly low prevalence of primary events. There was no significant difference in the time to event for the atorvastatin and placebo arms (the medians [IQRs] were 18 [12–24] months and 15 [4–32] months, respectively, *p* = 0.49, Mann–Whitney test).

The observation of lower lipid values during high disease activity, created a controversy regarding a possible inverse correlation between cholesterol and CVD risk in RA and, accordingly, the benefit of lipid lowering therapy on CVD risk reduction (58). The latter was examined comparing the associations between LDL-C reduction and cardiovascular outcomes in two large cohorts comparing 1,522 RA subjects to 6,511 general controls (GC) and 1,746 RA subjects to 2,554 patients with osteoarthritis, all of whom had dyslipidemia and were treated with statins for primary CVD prevention (59). Effective reduction of LDL-C was associated with a 29 and a 50% decrease in cardiovascular events in the RA/GC and RA/osteoarthritis cohorts, respectively. Despite the fact that the prevalence of traditional CVD risk factors was higher in RA patients, the decrease in cardiovascular events arising from lowering LDL-C concentrations were consistent in RA groups and their matched controls, highlighting the non-inferiority of statins in CVD management in RA.

Among 430 RA patients (181 statin-exposed and 249 statin-unexposed) without previous cardiovascular event, statins induced a 16% decrease in TC levels and were associated with a significant CVD risk reduction (adjusted HR 0.45, 95% CI: 0.20–0.98) and all-cause mortality (adjusted HR: 0.43, 95% CI: 0.20–0.92) (60). Comparable results were reported regarding the reduction of TC levels (15%) after statin intervention for secondary prevention. However, in this smaller group consisting of 78 RA patients, statins failed to offer a significant protection on CVD and all-cause mortality (HR: 0.58, 95% CI: 0.07–4.79 and 0.79, 95% CI: 0.18–3.53, respectively) (60).

Current guidelines encourage the early initiation of statins and the adoption of strict LDL-C treatment goals after the incidence of myocardial infarction (MI) (61, 62). As a result, in contrast to primary CVD prevention in RA, where a decision for statin treatment is complicated by a rather elaborate assessment of cardiovascular risk, treatment options after primary cardiovascular events could be considered more specific. However, a retrospective study indicated lower initiation of statins (HR: 0.69, CI: 0.58–0.82) following MI in 877 RA patients compared to 66,107 controls (63). Furthermore, a higher statin discontinuation was reported in the RA group. The observed unwillingness of physicians to initiate statins for post-MI treatment as well as the lower statin adherence may be attributed to a higher concern for adverse events, such as hepatotoxicity and myopathy, due to the preexisting therapy of RA patients with DMARDs combined with lower cholesterol levels compared to non-RA subjects. As no evidence exist confirming such safety concerns, the non-administration of the evidence-based medication for secondary CVD prevention seems highly irrational. Data regarding the associations between statin therapy and cardiovascular benefit in this indisputably high-risk patient group are limited.

Secondary CVD prevention, with either atorvastatin 80 mg or simvastatin 20–40 mg, was comparable across 87 patients with RA and 8,801 controls, followed for 4.8 years after MI in the Incremental Decrease in Endpoints through Aggressive Lipid lowering (IDEAL) study (64). Cardiovascular events occurred in 26.4% of RA patients and 28.7% of controls (*p* = 0.70), while no difference was noted considering the lipid lowering effect of treatment, although lower baseline TC and LDL-C values were recorded in the RA group. In addition, a *post hoc* analysis of the IDEAL trial and data provided by the Treating to New Targets study, also examined the effect of intensive statin therapy on secondary composite cardiovascular outcomes. The study reported a 20% cardiovascular risk reduction with atorvastatin 80 mg in both patients with and those without inflammatory joint disease, compared to less intensive statin treatment (65). However, despite the large number of patients (18,889 patients of whom 199 with RA, 46 with ankylosing spondylitis, and 35 with psoriatic arthritis), the decrease in cardiovascular events through intensive lipid lowering therapy was not significant for patients with inflammatory joint disease. The incidence of cardiovascular events was 37/156 and 35/124 in the intensive and non-intensive statin treatment group, respectively (HR: 0.81, 95% CI: 0.51–1.28).

The general consensus is that statin treatment in RA is not inferior regarding both the achievement of lipid targets and cardiovascular risk management, compared to the general population. To provide additional support, poor compliance in RA patients already assigned to statin treatment was associated with 67% increased risk of acute MI (HR: 1.67; 95% CI: 1.24–2.25), regardless of the timing of first statin prescription or prior CVD status (66). Last but not least, no significant difference in the frequency of adverse events has been reported in RA patients, while considerations on possible negative interactions between statins and DMARDs were not confirmed in large cohort studies (67, 68). Trials evaluating the efficacy of statins in CVD management in RA are summarized in Table 2.

**Table 2:** Controlled trials on the efficacy of statins on cardiovascular risk management in rheumatoid arthritis.

Reference	Population	Hypothesis assessed	Primary end point	Outcome
Kitas et al.—TRACE-RA (57)	2,986 RA patients	Atorvastatin 40 mg inferior to placebo	A composite of CVD death – Non-fatal MI – Cerebrovascular accident – Transient ischemic attack – Hospitalized angina – Coronary and non-coronary revascularization	– HR: 0.66 *p* = 0.119 NS (95% CI: 0.40–1.11) – Greater LDL reduction in atorvastatin group Early termination of trial

An et al. (59)	1,522 RA patients–6,511 GN (RA/GN cohort)	Effective LDL reduction-CVD treatment with – Atorvastatin 10–80 mg – Simvastatin 5–80 mg – Fluvastatin 20–80 mg – Lovastatin 10–40 mg – Rosuvastatin 5–40 mg – Pravastatin 10–80 mg	– MI – TIA – Angina – Stroke – Intermittent claudication – Heart failure Death from CV disease	Lowered LDL vs. not lowered – HR: 0.71 in RA/GN (95% CI: 0.57–0.89) – HR: 0.50 in RA/OA (95% CI: 0.43–0.58)
	1,746 RA patients –2,554 OA (RA/OA cohort)			Similar reduction of CV events in RA and controls – HR: 0.67–0.68 for RA – HR: 0.72 for GN – HR: 0.76 for OA

Sheng et al. (60)	Primary prevention – 430 RA patients (181 statin-exposed) – 1,269 OA patients (696 statin-exposed) – Secondary prevention – 78 RA patients (60 statin-exposed) – 247 OA patients (175 statin-exposed)	Effectiveness of statins on TC, CV risk, and mortality in RA and OA patents in terms of primary and secondary prevention	– TC change from baseline – CV events – All-cause mortality during follow-up	– In RA primary prevention:Reduced CV events – HR: 0.45 (95% CI 0.20–0.98)Reduced all-cause mortality – HR: 0.43 (95% CI 0.20–0.92)16% decrease in TC – In RA secondary protection:NS decrease in CV events – HR: 0.58 (95% CI 0.07–4.79)NS decrease in mortality – HR: 0.79 (95% CI 0.18–3.53)15% decrease in TC

Semb et al. —IDEAL (64)	87 RA patients–8,801 general population	Effectiveness of statins on lipid levels and CV in *secondary prevention* in RA – 39 atorvastatin 80 mg48 simvastatin 20–40 mg	CV event – Angina pectoris and/or non-fatal and fatal acute MI – Heart failure – PCI or CABG – Stroke or TIA	– Comparable reductions in lipid levels – CV events in 23/87 (26.4%) of RA patients vs. 2,523/8,801 (28.7%) in general population (*p* = 0.7) – NS difference between atorvastatin and simvastatin group (*p* = 0.24)

Semb et al. (65)	– 199 RA patients – 46 AS patients – 35 PsA patients 18,609 controls	Effectiveness of statins on CV events in *secondary prevention* – Atorvastatin 10 or 80 mg Simvastatin 20–40 mg	CV event – Angina pectoris and/or non-fatal and fatal acute MI – Heart failure – PCI or CABG – Stroke or TIA	– Atorvastatin 80 mg led to a 20% CVD reduction in both patients and controls compared to low-dose statin treatment—NS(*p* = 0.36) – Comparable decrease in lipid levels

De Vera et al. (66)	4,102 RA statin users	Impact of statin discontinuation CVD and all-cause mortality	CVD mortality and all-cause mortality	– HR: 1.60 (95% CI 1.15–2.23) for CVD mortality – HR: 1.79 (95% CI 1.46–2.20) for all-cause mortality

*RA, rheumatoid arthritis; CVD, cardiovascular disease; MI, myocardial infarction; HR, Hazard ratio; CI, confidence interval; NS, non significant; GN, general controls; OA, osteoarthritis; LDL, low-density lipoprotein; TC, total cholesterol; CV, cardiovascular; PCI, percutaneous coronary intervention; CABG, coronary artery bypass graft; TIA, transient ischemic attack; AS, ankylosing spondylitis; PsA, psoriatic arthritis*.

Ideally, more prospective, large controlled trials investigating the impact of statins on hard cardiovascular outcomes in RA patients are needed. Ethical considerations, and the very large sample sizes and long follow-up periods required to reach statistical significance, make such trials extremely difficult. Therefore, a reevaluation and elaborate approach of existing data becomes more necessary in order to redefine the role of statins in CVD management in RA.

## Disease Activity

**Figure 1:**
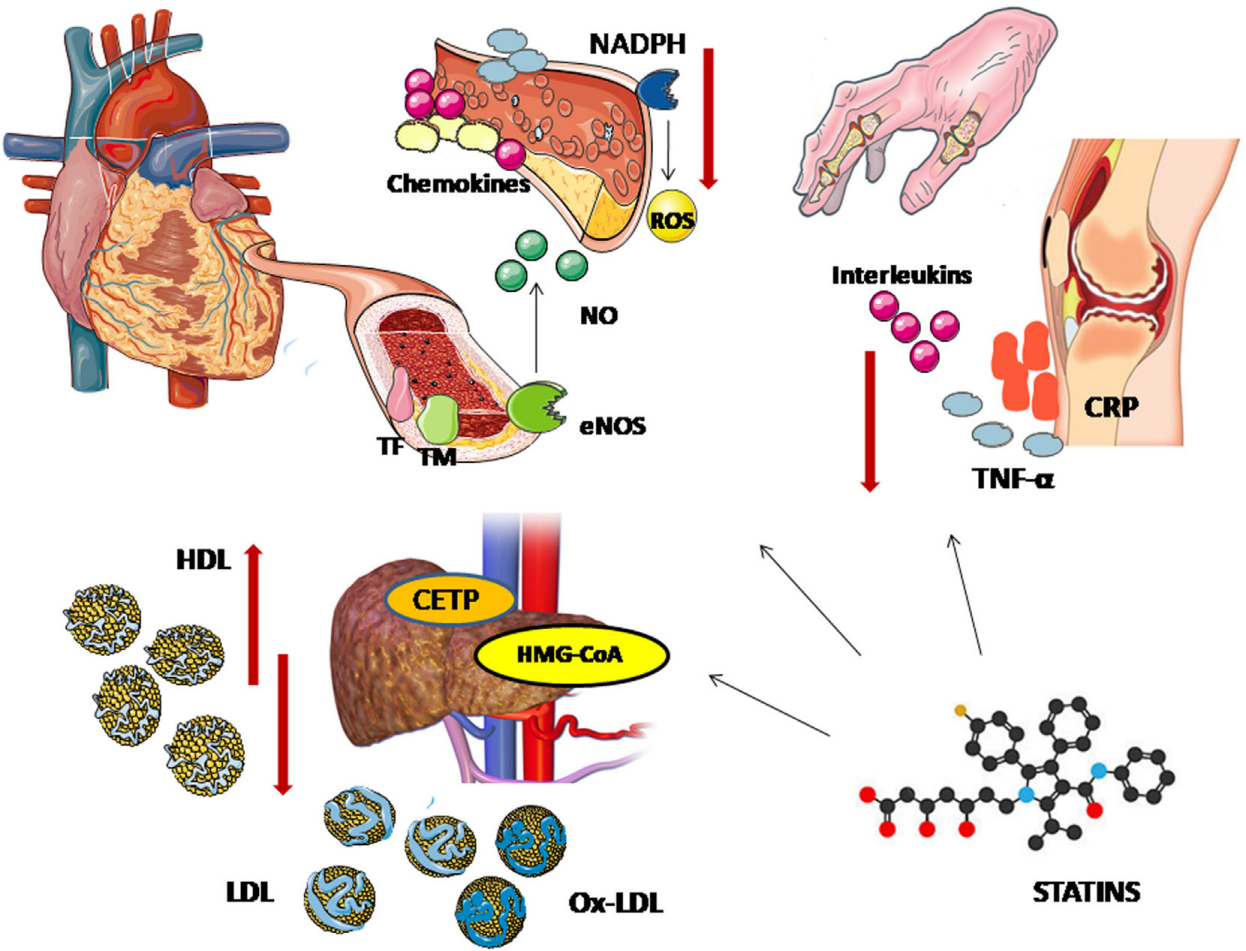
Pleiotropic effects of statins. The lipid lowering effects of statins is attributed to their action on 3-hydroxy-methylglutaryl coenzyme A (HMG-CoA reductase). Statins block the pathway for synthesizing cholesterol in liver by competitively inhibiting HMG-CoA reductase, the rate-controlling enzyme of the mevalonate pathway, leading to lower circulating low-density lipoprotein (LDL) cholesterol levels. A decrease in cholesteryl ester transfer protein (CETP) results in a modest increase in apolipoprotein A-I and high-density lipoprotein (HDL) cholesterol levels. Through the upregulation of endothelial nitric oxide synthase (eNOS), statins promote nitric oxide production and enhance endothelium-dependent vasodilatation. Statins also modulate the endothelial expression of cytokines, chemokines and leukocyte adhesion molecules, decreasing vascular inflammation—an important contributor to the vascular atherogenetic process. Furthermore, affecting both the endothelial production of inflammatory factors and cholesterol uptake, statins stabilize the atheromatic plaques. Their benefit on vascular function is also associated with the downregulation of nicotinamide adenine dinucleotide phosphate (NADPH) which results in lower levels of reactive oxygen species (ROS). Their antioxidative effects are also reflected on a decrease in oxidized LDL (oxLDL) levels. Statins elicit downregulation of tissue factor (TF) and overexpression of thrombomodulin, showing antithrombotic properties. Finally, the potential systematic beneficial effect of statins on systemic inflammatory diseases can be attributed to their ability to reduce inflammatory cytokines, such as tumor necrosis factor-α (TNF-α), interleukins, and C-reactive protein (CRP).

Although in the general population the cardioprotective effect of statins is almost entirely attributed to their lipid-lowering properties, there is some evidence supporting that their therapeutic properties expand beyond these and may be particularly elevant in inflammatory states such as RA. Their so-called pleiotropic effects include anti-inflammatory, antiproliferative, antithrombotic, antioxidative, and immunomodulatory properties (69) (Figure 1). Hence, there is growing interest regarding their potential influence on the inflammatory process characterizing autoimmune diseases. Beside their administration as cardiovascular risk modifiers, attempting role as adjuvant therapy for the control of inflammation in RA has stimulated experimental and clinical research.

In a study of 118 RA patients on DMARD therapy, atorvastatin 40 mg provided a significant improvement on disease activity score 28 (DAS28) and acute-phase reactants compared with placebo after 6 months of treatment (41). Greater response rates were also recorded in the atrovastatin group versus placebo, among 111 patients receiving the oral Janus kinase inhibitor, tofacitinib (70). Mowla et al. confirmed a suppression of disease activity, as evaluated by inflammatory markers and clinical assessment after three months treatment with atovastatin 40 mg as an adjunct to current disease-modifying antirheumatic drugs (71). A recent meta-analysis of 11 relevant studies reported a standardized mean difference in DAS28 of −0.55 (95% CI: −0.83 to −0.26, *p* = 0.0002), with an *I*^2^ value of 68%, documenting the positive effect of statins on RA activity (72). Interestingly, statin treatment tended to be more beneficial in those cases with higher disease activity. However, the observed association is to be critically evaluated, as no adjustment for DMARD therapy was obtainable in the analysis, due to high heterogeneity of DMARD use among the included studies.

The contribution of statins to the improvement of clinical aspects of RA could be attributed to their ability to affect multiple steps in the pathogenesis of the disease. Chronic joint inflammation results from the overexpression of several inflammatory mediators, including cytokines, growth factors, adhesion molecules and T lymphocytes (73). Given that TNF-α and ILs are key factors in the process of chronic arthritis (74, 75), the ability of statins to decrease such mediators may mediate an anti-inflammatory benefit in RA (76). Simvastatin treatment has been found to decrease TNF-α levels in RA patients, accompanied by reduction of serum markers of inflammation (77). In that respect statins proved effective in diminishing CRP levels in the general population, along with a recorded suppression of inflammatory mediators, such as IL-6 (78). In line with such observations a recent meta-analysis confirmed the favorable outcome of statins on several clinical parameters of RA such as erythrocyte sedimentation rate, CRP, tender, and swollen joint count, highlighting the potential dual benefit of statins on both joint and vascular inflammation (79).

On the other hand, the reduction in CRP levels following statin treatment might not necessarily be accompanied by improvement in the overall disease status, as shown in a study evaluating the effects of 6-month rosuvastatin therapy in 50 RA patients (80). In contrast to CRP, a trend toward worsening in IL-6 levels in the rosuvastatin group in the same study suggested a rather direct effect of statins on CRP liver production rather than a global suppression of inflammation.

The impact of simvastatin on RA disease activity was evaluated in a cohort study, including 100 RA patients under DMARD treatment. With respect to recommendations for statin treatment, 50 patients were chosen to receive simvastatin 20 mg/day additionally to the existing DMARD therapy, forming the intervention group. Simvastatin treatment for 6 months resulted in a more pronounced, but not statistically significant reduction in CRP levels, DAS28, early morning stiffness and tender joint count compared to the control group. In contrast to the clinical and biological variables, the global assessment of disease activity performed by a non-blinded evaluator was the only parameter that significantly differed between the two groups after 6 months (difference between groups: −9.54; 95% CI: −15.913 to −3.184; *p* = 0.007) (81).

Most of the observations concerning the anti-inflammatory effects of statins were made on study populations already receiving a multitude of disease suppressing drugs, so any impact on systemic inflammation cannot be attributed solely to a statin effect. Although some of the evidence points to a potential anti-inflammatory statin effect in RA, this “additional” effect has not been consistent or accurately quantified and its clinical significance (in the context of high-grade rheumatic inflammation) remains undetermined. However, the lack of significant adverse events in RA patient cohorts receiving several other potentially hepatotoxic medications is reassuring and their significant clinical effect at reducing cardiovascular risk make them an attractive choice in suitable patients.

## Conclusion

Optimal cardiovascular risk management remains a challenging theme in RA. Inflammation and primary atherosclerosis are highly associated and cannot be treated as independent factors. Statins have a beneficial impact on RA-related cardiovascular risk, due to their lipid lowering but also possibly angioprotective, antioxidative and anti-inflammatory effects. This, and their safety profile, makes them an ideal choice in the overall armamentarium aimed at complete control of RA disease and associated vascular morbidity. Despite this, the blanket use of statins on all RA patients cannot be supported by evidence, thus appropriate CV risk assessment tools for this population are necessary, which in addition to established risk factors could include non-invasive vascular assessments, particularly carotid IMT (82). Whether any anti-inflammatory statin effect is clinically important in the context of RA activity outcomes is intriguing and needs to be further evaluated in studies designed specifically for this purpose.

## Author Contributions

SS: literature search and writing of the manuscript. EN: design and revision of the manuscript. TD: literature search, writing, and revision of the manuscript. GK: design and final revision of the manuscript.

## Conflict of Interest Statement

The authors declare that the research was conducted in the absence of any commercial or financial relationships that could be construed as a potential conflict of interest.
